# Telemedicine and Haemodialysis Care during the COVID-19 Pandemic: An Integrative Review of Patient Safety, Healthcare Quality, Ethics and the Legal Considerations in Singapore Practice

**DOI:** 10.3390/ijerph19095445

**Published:** 2022-04-29

**Authors:** Sabrina Haroon, Teck Chuan Voo, Hillary Chua, Gan Liang Tan, Titus Lau

**Affiliations:** 1 Division of Nephrology, National University Hospital Singapore, Singapore 119228, Singapore; titus_lau@nuhs.edu.sg; 2 Centre for Biomedical Ethics, Yong Loo Lin School of Medicine, National University of Singapore, Singapore 117597, Singapore; mdvtc@nus.edu.sg; 3 Faculty of Law, National University of Singapore, Singapore 259776, Singapore; h_chua@nus.edu.sg; 4 Department of General Medicine, Sengkang General Hospital, Singapore 544886, Singapore; tan.gan.liang@singhealth.com.sg

**Keywords:** telemedicine, haemodialysis, COVID-19

## Abstract

The COVID-19 pandemic has been an unprecedented health crisis for the general population as well as for patients with chronic illnesses such as those requiring maintenance dialysis. Patients suffering from chronic kidney disease requiring dialysis are considered a high-risk population. Multiple reports have highlighted an increased need for intensive care and higher death rates among this group of patients. Most maintenance dialysis patients are in-centre haemodialysis patients who receive treatment in shared facilities (community dialysis centres). The inability to maintain social distancing in these facilities has led to case clustering among patients and staff. This poses a substantial risk to the patients, their household members, and the wider community. To mitigate the risks of COVID-19 transmission, telemedicine was rapidly adopted in the past year by nephrologists and other allied-health staff to provide care via remote consultations and reviews. Telemedicine poses unique challenges even in an era where so much is performed online with a high degree of success and satisfaction. In applying distant clinical care for maintenance haemodialysis patients via telemedicine, there is a need to ensure adequate protection for the health and safety of patients as well as understand the ethical and legal implications of telemedicine. We discussed, in this article, these three core aspects of patient safety and quality, ethics and legal implications in telemedicine, and how each of these is crucial to the safe and effective delivery of care in general as well as unique aspects of this in Singapore.

## 1. Introduction

Telemedicine is a well-established care delivery format in today’s health services structure [1]. Telemedicine is generally defined as the assessment of health, diagnosis, treatment, interventions, or any health-related care activities that are provided over a distance using info-communication technology [2]. It was first intended to provide better and easier healthcare access to remote (usually small) populations [3,4]. Later, as technology matured and mindsets shifted, the adoption of telemedicine became more widespread and was no longer restricted to areas that were hard to reach [5]. It has since evolved to become a complementary option of care delivery that fulfils many important key measures of healthcare services such as optimised efficiency, increased convenience, and cost lowering without compromising patient safety and satisfaction [6,7]. Over the years, and especially in the last decade, there has emerged an even broader umbrella of digital healthcare solutions known as technology-enhanced care that offers even more possibilities [8]. The advancement of electronic and mobile technologies has made possible the implementation of telemedicine in many fields of medicine [9]. The adoption of technology has permitted care to be delivered more efficiently and effectively in the home environment and removed the dependence on health facilities.

In addition to its adoption as part of day-to-day healthcare, telemedicine has useful applications as a disaster response tool [10]. There are several categories of health applications in disaster response, including ambulatory and primary care, specialty consultation, remote monitoring, triaging, and medical logistics coordination. Telemedicine is described as a force multiplier, providing medical and public health expertise at a distance, and minimizing the logistic and safety issues associated with on-site care provision [11]. A telehealth white paper prepared by a telehealth think tank group from the Office for the Advancement of Telehealth, Health Resources and Services Administration in the United States elaborated on the strategic applications and challenges of how telemedicine can be an effective component of disaster response. The group concluded with recommendations that include shifting mindsets, integrating telemedicine into disaster response plans, developing an inventory of telehealth resources and protocols, provision of necessary telehealth equipment and software, and hardening telehealth networks to ensure reliability [11].

Logically, telemedicine is also ideal for managing a global infectious disease pandemic. It has several key qualities that effectively enhance the emergency response during an infectious disease outbreak such as the current COVID-19 pandemic. A key factor in slowing the transmission of an infectious pathogen is “social distancing” to decrease person-to-person contact. Telemedicine can perform remote triaging of care, assist in diagnosis via video consultations and testing, provide care for those who are infected, disseminate public advice and education, and serve as a tool to coordinate and deliver routine medical care [12]. The implementation of telemedicine during the ongoing COVID 19 pandemic was not only restricted to first-world economies in North America and Europe but lower-resource countries such as Africa, Latin America, the Middle East, and Asia [9,13]. These countries have utilized telemedicine for COVID-19-related and non-COVID-19-related medical conditions in their health services. All of them reported satisfactory outcomes, and telemedicine generally benefitted the population.

In Singapore, as elsewhere, the COVID-19 pandemic has accelerated the adoption of telemedicine nationwide [14]. In 2020, at the height of the pandemic, strategic measures were taken to escalate various domains of telemedicine, particularly telemonitoring and telesupport. These include tools such as the Singapore COVID-19 Symptom Checker, where artificial intelligence is used to advise users on their next course of action should they experience the symptoms listed in the application. The “Flu Go Where” microsite advises those who meet the criteria for COVID-19 screening on the location of the nearest clinic. A group in Bern, Switzerland, has also recently published a framework for online forward triaging and cited seven criteria that will ensure the adequacy of the tool used for this purpose [15]. In terms of pharmaceutical logistics, a similar service, the “Mask Go Where” webpage, advised on mask distribution. In contrast, medication delivery services were ramped up to deliver medication nationwide, avoiding queues and healthcare facility visits. Every adult resident received a complimentary pulse oximeter to monitor oxygen saturation, and telekiosks for video consultations were made available as part of a broader comprehensive health support plan [16]. Telemedicine was also deployed in managing infected patients at community care facilities and community recovery facilities [17,18]. Towards late 2021, more than 80% of the Singapore population were fully vaccinated, and many of those infected had very mild diseases and were successfully managed at home using teleconsult and telemonitoring [19].

As part of the overall COVID-19 management strategy, Singapore’s Ministry of Health (MOH) encouraged the use of telemedicine for all “non-essential” healthcare services in order to reduce hospital workloads and promote safe distancing [20]. Dialysis services were deemed essential, but the treatment of other renal diseases with no active or recurrent symptoms was classified as “non-essential” and suitable for teleconsultation [21]. Dialysis services generally encompass two broad areas—the dialysis treatment that is conducted at the facility and the care and review of dialysis patients. In-centre haemodialysis (ICHD) will require patient attendance at the facility, but the clinical review of ICHD patients is an activity that can potentially be conducted off-site via telemedicine. One of the main infection control measures at the start of the pandemic was to control the movement of healthcare professionals to minimise potential exposure and spread. This article describes the transition and structure of providing care to ICHD patients in Singapore via telemedicine platform and shares our insights on this model of remote care from the perspective of clinical quality, ethical standards, and legal considerations.

## 2. Management of ICHD Patients

**Figure 1:**
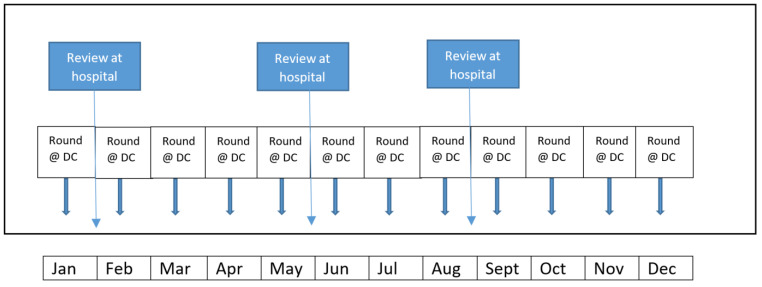
Structure of review at the dialysis unit before the COVID-19 pandemic.

Singapore has seen an increasing number of renal failure patients over the years. The Singapore Renal Registry registered 8211 prevalent dialysis patients in 2020 [22]. Most of the patients (86%) are ICHD patients receiving haemodialysis treatments from over 100 satellite dialysis units in Singapore [22]. These are either charity-funded units or private for-profit units. The management of ICHD patients in Singapore generally involves two nephrologists. The dialysis centre nephrologist is tasked with ensuring that patients in a particular centre are doing well and managed optimally. Hence, it is mandatory that all ICHD patients are reviewed at least once a month at the dialysis centre [23]. The primary nephrologist at the hospital complements the monthly (shorter) review and sees the patient at the clinic (when the patient is not on dialysis) for a lengthier consult to have an in-depth discussion and a detailed physical examination. The typical schedule is every four to six months (Figure 1). Given this structure of practice, both will jointly manage the patients and exchange management decisions via email or phone call.

The dialysis centre nephrologist will review the patient while the patient is on haemodialysis (HD) treatment, focusing on more immediate dialysis issues (such as access to health, fluid, and blood pressure management, anaemia correction, and metabolic bone disorder). Physical examination is brief and mainly focused on volume assessment or other simple examinations that can be done with the patient seated on a recliner chair. A detailed physical examination is difficult to conduct, given that ideal positioning and manoeuvres are not possible while on dialysis. Other limitations of a review conducted during HD treatments are that some patients may be symptomatic on dialysis, with multiple ongoing alarms and proximity to the next patient in an open cubicle. Therefore, some of the clinical problems cannot be fully addressed. These unresolved clinical issues and other discussions are then referred to their primary nephrologist at the hospital. Patients will have more privacy in the consulting room and when a detailed physical examination can be performed. Despite the stated limitations, dialysis centre reviews are still a very effective adjunct to care for these patients as it does not require travel or frequent additional costly hospital appointments.

## 3. Conduct of Dialysis Review via Telemedicine

Telemedicine has been widely used in managing home dialysis patients in many parts of the world (both home haemodialysis and peritoneal dialysis) [24,25]. MOH Singapore has published the National Telemedicine Guidelines (2015) for local healthcare practice [26]. However, there is no specific guidance for telemedicine at the dialysis centre. As a result, the use of telemedicine in satellite dialysis centres had been limited until the COVID-19 pandemic necessitated a change in approach. The small geographical footprint of this island-state and the long-held tradition that in-person review is the standard of care have also largely contributed to this lack of adoption of telemedicine in ICHD care. During the initial phase of the pandemic in Singapore, including the “circuit breaker” period (i.e., a nationwide partial lockdown), vaccines were non-existent, and the transmissibility or “R-Naught” was not entirely clear. As such, many satellite dialysis centres incorporated telemedicine as part of dialysis care to reduce the need for physical review by the nephrologist. Given the different types of patient reviews and intended purpose, telemedicine is used primarily to replace the monthly in-person review at the dialysis centre. It should be noted that most of our local nephrologists have multiple sites of practice that include a hospital and more than one satellite dialysis centre (usually up to four centres). In-person nephrologist visits to the satellite dialysis centres may still inadvertently lead to outbreaks arising from interactions between the nephrologists, staff, and patients at the centre. However, there are strict infection control measures, including the use of full personal protection equipment (PPE).

**Figure 2:**
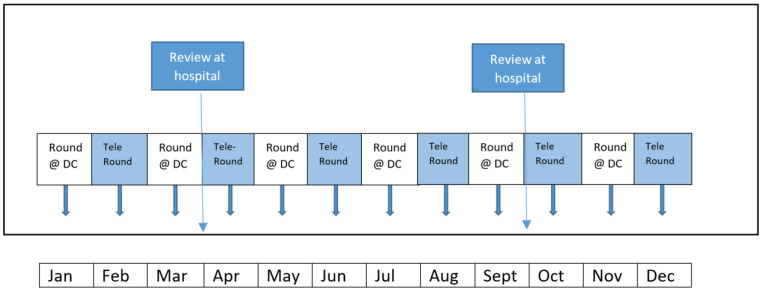
Structure of review at the dialysis unit since onset of the COVID-19 pandemic.

As the COVID-19 outbreak has entered into an endemic phase in recent months, restrictions on the movement of healthcare professionals between healthcare facilities have been reduced. However, telemedicine is still favoured by some to reduce the frequency of in-person physician reviews at the dialysis centre. Most dialysis centres have a fixed schedule for blood and adjunct tests such as bioimpedance and vascular access flow monitoring that is performed every two months. The nephrologist will usually perform a physical in-person round during blood test month as there are often discussions about medications, referrals, and various changes. Telemedicine review is then conducted on non-blood test months, with the option to alternate between physical reviews at the dialysis centre. In addition to these reviews, the primary nephrologist will continue to have in-person consults at the hospital’s clinic (Figure 2).

## 4. Ensuring Patient Safety and Maintaining Care Quality

**Figure 3:**
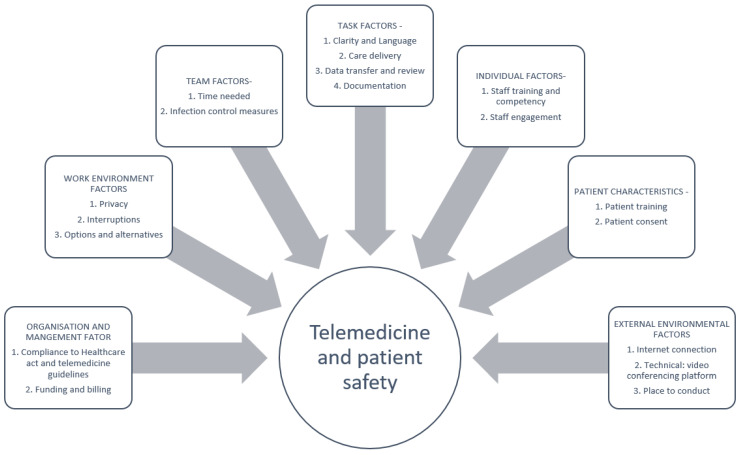
Core elements of patient safety while adopting telemedicine.

The COVID-19 pandemic has certainly challenged many healthcare systems. To respond to the crisis, these systems have had to reorganise instantly, risking possible threats to patient safety, and compromising the quality of care [27,28]. As we increase our reliance on telemedicine during the COVID-19 pandemic, we must also be cognisant of the potential gaps that may exist. New technologies and care models come with attendant risks [29]. Healthcare systems adopting telemedicine should focus on pre-emptive risk reduction with adequate planning and emphasis on details. There are seven key elements as described by Vincent that influence patient safety, and these should be taken into consideration when adopting telemedicine [30]. The components within each of these elements related to the conduct of ICHD rounds via telemedicine are shown below (Figure 3).

Telemedicine for the review of ICHD patients in Singapore takes the form of real-time interactive video conferencing between the nephrologist, the dialysis centre nurse, and the patient. As there are no established telemedicine platforms amongst dialysis providers in Singapore, most choose to use Zoom, a commercial third-party video conferencing platform that was not specifically designed for healthcare use. It was simple to use but with few enhanced features to support better clinical review of patients. Some nephrologists also adopted the “store and forward” method of telemedicine by getting the dialysis centre to send relevant data that is usually needed for the review of ICHD patients, such as dialysis treatment charts, recent laboratory results, and measurements done at the dialysis centre. The live teleconsult would be scheduled soon after such information had been obtained and reviewed by the nephrologist. Some dialysis providers have a software system that allows the nephrologist to access the system and view relevant information directly. Others who do not follow this pattern of practice will review all patient information during the live teleconsult (with the attending nurse updating the nephrologist).

One critical aspect of care for HD patients is maintaining dialysis access. In our effort to reduce direct physical examination during the pandemic, telemedicine is a useful alternative for access evaluation. Findings of aneurysms, infections, depigmentation, skin thinning, ulcerations, oedema, and catheter exit site issues can all be detected remotely [31]. Supplementary information relevant to access patency (such as access flow measurement, arterial and venous pressure changes during treatment, stability of prescribed blood flow) can be easily captured and transmitted via electronic means [32].

It is crucial to understand that telemedicine will not entirely reduce the acquisition or transmission of COVID-19. However, it does help mitigate the risk along with many other appropriate infection control measures. Hence, it is a positive effort toward patient safety. In adopting telemedicine, we need to ensure no compromise on patient care by omitting the in-person physical review [33]. The standard of care and expected tasks must be fully accomplished. There should be sufficient time allocated for the nephrologist, staff, and patient to deal with problems usually addressed during physical rounds. Most of the evaluation, such as routine blood tests, dialysis parameters, medication lists, vascular access measurement, and bioimpedance, can be reviewed either using an online dialysis software or through the transfer of data. What is important is to ensure that the information provided for a teleconsult is what is otherwise provided for an in-person physical review. The data should be made fully available to the nephrologist and presented in the same way that is easily interpretable by the nephrologist performing the teleconsult. Further, there should be an established process workflow to document the review (via telemedicine) for the healthcare team to follow up on any changes. To reduce the risk of missing any clinical findings that otherwise would have been detected during physical rounds, dialysis nurses can receive additional targeted education and training on simple examinations [34]. Suppose the patient is unwell and requires a physical review for better assessment. In that case, rapid coordination of care using a mutually agreed pathway with the primary nephrologist must be in place and escalation protocols established.

Beyond the clinical processes required to deliver high-quality care, the infrastructure needed to make this possible cannot be undermined. Access to a robust and effective digital infrastructure is critical in the delivery of care via telemedicine. In the context of Singapore’s ICHD telemedicine initiative, a laptop with a high-quality audio-visual function was the main device for connecting the nephrologist with the patient at the dialysis centre. There were times when a tablet or a mobile device was used when a laptop was not available. Ideally, the hardware must be tested before implementation to ensure sufficient clarity for a teleconsult to take place. A specially designed software package that fits dialysis care or a software system that integrates with the hospital’s electronic health record would be advantageous. However, this was not possible in Singapore at the time of adoption due to the rapidity of emergent adoption in this situation. The stability and bandwidth for transmission are generally excellent in Singapore and do not pose a problem. The infrastructure for telemedicine must be available when needed, and equally, the technology must be reliable and secure, as is discussed further in the “Cybersecurity and Data Privacy” section below.

As telemedicine in the ICHD setting is new to local practice, the monitoring of various aspects of care will be necessary to ensure satisfactory clinical outcomes and the safe delivery of care (Table 1). One preliminary study involving the largest charity dialysis organisation in Singapore reported that telemedicine was a successful tool for physician oversight under movement control measures implemented during the COVID-19 pandemic. There was no increase in hospitalisation or mortality after the implementation of telemedicine as a remote form of patient review [35]. Despite this encouraging report, there is still a need to evaluate the workflow and make further adaptations. Apart from the clinical outcome (as published), monitoring of safety and quality should follow the Donabedian model that categorises a healthcare system into structure, process, and outcome (Figure 3). Incidents can be reduced by designing new processes with a focus on human factor engineering principles, coupled with frequent evaluations during the initial phase and to implement suggested changes. An example commonly used is the PDSA cycle, Plan-Do Study Act [36]. When an adverse event or a near miss occurs, a rapid cycle root cause analysis or concise incidence analysis should be conducted soonest to improve the structure or process [37].

Given that this may involve a significant change in the structure and processes, understanding staff and patient satisfaction will be necessary [38,39]. Patient satisfaction is part of patient-centric care that is championed as one of the aspirational core values of the healthcare system in Singapore. Staff satisfaction is also essential as it does have a significant association with quality of care. The quality indicators for outcome monitoring should include hospital admissions and specifically for fluid overload, as volume excess is usually diagnosed during the physical review. Other issues resulting in hospital admission should be reviewed. In addition, an effort made to identify if any of the admissions can be avoided if a physical review was performed in place of the teleconsult. Specific to our local context, over time, we aim to determine the appropriate mix of physical reviews and teleconsults to ensure that the care of our ICHD patients is not compromised [40].

## 5. Ethical Considerations and Justifications

The ethicality of introducing or increasing the reliance on telemedicine in the management and care of ICHD patients during a pandemic like COVID-19 depends on whether it would help achieve public health goals. The section on telemedicine in the 2016 edition of the Singapore Medical Council Ethical Code and Ethical Guidelines (ECEG) provides guidance for the responsible provision of telemedicine. However, it does not elaborate on its use in the context of a healthcare crisis [41]. At the beginning of the pandemic, there were many scientific unknowns. As a result, it was hard to assess the extent to which telemedicine, as a mode of physical distancing between physicians and patients, would help to reduce spread, prevent mortality and morbidity, or reduce the strain on the health system by protecting healthcare workers. Nevertheless, the use of telemedicine, in conjunction with other public health measures (as mentioned above), is justifiable by the precautionary principle [42], which directs policymakers to introduce measures to protect populations against reasonably foreseeable serious risks in the absence of absolute certainty.

Proportionality [43] is another important consideration: the anticipated public health benefits that accrue from the intervention should be proportionate, that is, significantly outweigh the expected burdens, costs, and harms to patients as well as to the health system. Singapore is one of the most wired and digitally inclusive countries in the world with a highly advanced information and communication technology (ICT) infrastructure [44,45]. Thus, the implementation of teleconsults in satellite dialysis centres should not result in significant burdens on its health system, or on patients, especially for those whom online interactions have become a norm in many parts of their lives during the pandemic. As discussed earlier, there is a need to mitigate harm to patients by ensuring that telemedicine does not displace any necessary physical review. Such harm mitigation may be further secured by guidelines on the minimum frequency for which patients on maintenance dialysis ought to receive a physical review. Setting this minimum standard would also promote equity in terms of patient outcomes. Other than these considerations, the ethical acceptability of telemedicine for ICHD patients should also be guided by beneficence. It may be important for some patients to have more face-to-face encounters with their nephrologists to establish or maintain trust and a therapeutic doctor-patient relationship. Some patients may also face challenges engaging physicians during teleconsult due to their particular conditions (e.g., hearing impairment) [46]. Insofar as public health would not be compromised, these patients should be granted access to more in-person contact with physicians to ensure that their care is optimised.

This raises the question of the extent to which telemedicine, as a public health response to a pandemic, should be guided by a patient- or person-centred approach. The deployment of telemedicine to reduce risks of transmission and infection means that it cannot be highly tailored to the needs and preferences of individual patients. Respect for persons—as a core principle of patient-centred care—can be observed in other ways. This includes protecting patient privacy and the confidentiality of their medical information. As a function of respect for persons, patients should also be informed of the benefits and risks of telemedicine during the pandemic and the accompanying measures to ensure patient safety and care quality, which would also promote patient trust and acceptability of this modality of care. Failing to provide material information on telemedicine to patients would also have legal implications under the law of negligence, as discussed below.

As Singapore moves towards COVID-19 resiliency by achieving high population vaccination coverage and other public health responses, telemedicine may no longer be a necessary measure to mitigate public health risks. As has been argued, it is likely the case that health systems will continue to rely on telemedicine for ICHD patients and other patient groups because of its advantages for patients in terms of “flexibility, benefits and cost-saving” [47]. Compared to telemedicine for home dialysis patients, the advantages of telemedicine in these aspects for patients receiving treatment at dialysis centres are less clear. The ethical justification of telemedicine in a post-pandemic world largely depends on the balance of benefits for physicians (for example by reducing the frequency of physical reviews) the health system, and the benefits and potential harms for patients who could be assessed by both monitoring and relevant research on patient outcomes.

## 6. Regulatory and Legal Requirements

In a review of telemedicine frameworks across the globe during the COVID-19 pandemic, Singapore was identified as a “front-runner in Asia in terms of telemedicine adoption and healthcare system efficiency” [48]. This section provides an overview of Singapore’s laws, regulations, and guidelines regarding the use of telemedicine, as an example of how a national legal framework can bolster telehealth implementation and reinforce the recommended practices in patient safety and medical ethics that were discussed above.

First, MOH Singapore will be introducing a set of telemedicine regulations under the Healthcare Services Act 2020, which are expected to come into force at the end of 2023 [49]. This comes as part of a nationwide transition from a premises-based licensing regime to a services-based licensing regime for healthcare providers [50]. Second, the National Telemedicine Guidelines (2015) (NTG) stipulated the best practices for telemedicine services in four broad domains: clinical standards and outcomes, human resources, organisation, and technology and equipment [26]. Although the NTG has no force of law, MOH Singapore may decide to sanction a healthcare provider for non-compliance with the NTG [50]. The guidelines contain two key principles: (i) the overall standard of care for telemedicine must not be any less than what is provided in face-to-face services, and (ii) where a face-to-face consultation is not reasonably practical. Telemedicine is better than having no access to care at all. Third, telehealth products are regulated under the Health Products Act 2007. There are also official guidelines for telehealth products and artificial intelligence (AI) in healthcare [51,52]. Separate regulations and guidelines apply to telepharmacy [53,54].

Singapore has a clear framework for accountability and disciplinary action in relation to the use of telemedicine. This is in line with recommendations from the World Health Organization regarding strategies for implementing telemedicine during the COVID-19 pandemic and beyond [55]. In terms of medical professional ethics, the ECEG states that in telemedicine, physicians must endeavour to provide the same quality and standard of care as in-person medical care [41]. It is important for physicians to be aware of the principles of the ECEG concerning the responsible use of telemedicine and upholding patient confidentiality. A breach of the ECEG could result in disciplinary sanction for professional misconduct under the Medical Registration Act 1997 [56]. For example, one key principle in the ECEG states that physicians “must give patients sufficient information about telemedicine for them to consent to it” [41]. Therefore, it would be helpful to anticipate the limitations of using telemedicine for patient care (e.g., technological malfunctions or diagnostic limitations) and design standardised consent forms accordingly [57]. This could be supplemented with training and protocols for identifying which patients are suitable for teleconsultation and should cover escalation procedures to in-person care [57,58].

When diagnostic or treatment errors occur from telemedicine, the Singapore courts will assess whether the actions of the doctor in question can be supported by a responsible body of expert opinions that satisfies a threshold test of logic [59,60,61]. If so, the doctor will not be found negligent. However, suppose errors occur partly as a result of clinical negligence and also technological malfunction. In that case, the apportionment of liability between a clinician and a telehealth company (such as a software developer or manufacturer) will depend on the facts of each case [50]. A physician may remain liable for overseeing the choice and use of a telehealth modality even if technology is primarily to blame [52,62]. Regarding liability for not obtaining a patient’s informed consent, the Singapore courts will assess whether the physician disclosed “relevant and material” information to the patient about telemedicine, including risks and reasonable alternatives. What is “relevant and material” will be determined with reference to what the “reasonable patient” and “specific patient” would want to know [63]. Once Section 37 of the Civil Law (Amendment) Act comes into force, it will amend the law such that expert medical opinion shall be used to determine whether the standard for information disclosure has been met [64].

## 7. Cybersecurity and Data Privacy

Cybersecurity is an important consideration that has implications for patient safety, ethics, and the law. Data privacy and protection have been identified as a core challenge and critical factor for the success of telemedicine, which warrants a special discussion of this topic [65]. In the United States, the Office for Civil Rights at the Department of Health and Human Services issued a Notification of Enforcement Discretion for telehealth during the COVID-19 nationwide public health emergency. This has allowed American healthcare providers to provide telehealth services over platforms such as Skype and Zoom in good faith without facing penalties for violating regulatory requirements under the Health Insurance Portability and Accountability Act of 1996 (HIPAA) Rules [66]. In Singapore, there is no equivalent relaxation of data protection laws for telemedicine specifically, so healthcare providers should be mindful of the following rules.

The ECEG states that physicians “must take reasonable care to ensure confidentiality of medical information shared through technology and ensure compliance with any applicable existing legislation and regulations governing personal data” [41]. This would involve complying with cybersecurity protocols and choosing a reasonably secure third-party telemedicine platform. System vulnerabilities may result in inappropriate access to patient information, medical device malfunction, or a breakdown in healthcare services provided over remote communication platforms. These breaches can have profound effects on patient safety and care. Moreover, the principle of respect for persons is a further reason for giving due consideration to data security and protection regarding the online transmission or sharing of medical information.

On an organisational level, the Personal Data Protection Act 2012 requires healthcare providers to (i) obtain consent from patients to the collection, use, or disclosure of their personal data, unless a recognised exception applies; and (ii) make reasonable security arrangements to protect the data from unauthorised access, among other things [67]. Although there is an “Emergency Response Exemption” for the collection, use, and disclosure of personal data without consent for contact tracing and other COVID-19 response measures, the need to make reasonable security arrangements to protect personal data still applies [68,69]. In 2019, the Personal Data Protection Commission (PDPC) issued fines totalling SGD $1,000,000 against two public health bodies for lax cybersecurity measures, which had resulted in hackers accessing the medical data of 1.5 million patients. In its decision, the PDPC noted that medical data is “of a sensitive nature” and requires a higher standard of protection [70]. Details of recommended cybersecurity measures and policies for healthcare providers can be found in MOH Singapore’s Healthcare Cybersecurity Essentials Guidelines (2021) and AI in Healthcare Guidelines [52]. Section 27 of the Healthcare Services Act 2020 also requires licensed healthcare providers to implement prescribed safeguards to protect medical records from unauthorised access. Failure to do so is a criminal offence. Additionally, the use of telemedicine comes with a risk of identity fraud, such as when third parties masquerade as patients through the use of manipulated videos known as deepfakes. Therefore, it would be prudent to verify patients’ identities at the outset of each teleconsult, using methods such as two-factor authentication [71]. The introduction of telemedicine should not raise additional privacy concerns relative to physical rounds in dialysis centres.

Many of the above considerations are mitigated by the manner in which telemedicine is conducted for ICHD patient care. Existing patients are already known to the nephrologist, and new patients are all registered formally and known to be actual patients undergoing dialysis treatment. Data transfers are securely done via an independent platform (from that used for teleconsultations), and all files are locked with a unique password to prevent unauthorised access. The teleconsult is supervised on the patient’s side, and this is helpful in ensuring clarity of the exchanges as well as follow-up on the prescribed changes. Telemedicine used in ICHD is expected to pass the same level of legal scrutiny as any other telemedicine services in healthcare.

## 8. Conclusions

Telemedicine is certainly feasible and can be impactful in many ways. While it is true that the use of telemedicine in the treatment plan of outpatient dialysis patients in Singapore was born out of necessity due to the worldwide COVID-19 crisis, it has accelerated a serious discussion regarding the possible sequelae of telemedicine—both positive and negative. Professionally, we must strive to maintain a high standard of care that prioritises patient safety and clinical efficacy. A patient-physician encounter is not simply a means to complete a clinical task or solve a health issue, but it is a relationship that is deeply intimate. Hence, there still exists a distinct emotive difference between a virtual reality interaction and a face-to-face interaction. Ethical and legal compatibility and compliance are certainly prerequisites. A carefully crafted framework for governance of the various aspects of telemedicine will strengthen its adoption and lead to long-term sustainability. Future research focusing on monitoring the quality of care delivered and the impact of safety in telemedicine is needed. Given how technology has transformed our everyday lives over the past few decades, it is inevitable that our healthcare landscape will be equally influenced by it over time. Ultimately, no matter the format of interaction or the method employed in accomplishing our clinical duties, we have a fiduciary responsibility to act in the best interest of patients with due regard to their values and preferences.

## Figures and Tables

**Table 1:** Quality measures telemedicine in dialysis unit in the Singapore setting.

	Quality Measures
**Structure**	Number of review per year (physical and telemedicine)
**Process**	Staff satisfaction Patient satisfaction
**Outcome**	Hospitalization rate after adoption of telemedicine Hospitalization numbers from fluid overload
